# Guide to evaluating performance of prediction models for recurrent clinical events

**DOI:** 10.1186/s41512-025-00187-7

**Published:** 2025-03-17

**Authors:** Laura J. Bonnett, Thomas Spain, Alexandra Hunt, Jane L. Hutton, Victoria Watson, Anthony G. Marson, John Blakey

**Affiliations:** 1 Department of Health Data Science, University of Liverpool, Liverpool, L69 3GL UK; 2 Department of Statistics, University of Warwick, Coventry, CV4 7AL UK; 3 Phastar, London, W4 5LE UK; 4 Department of Pharmacology & Therapeutics, University of Liverpool, Liverpool, L69 7BE UK; 5 Medical School, Curtin University, Perth, WA 6102 Australia; 6 Respiratory Medicine, Sir Charles Gairdner Hospital, Perth, WA 6009 Australia

**Keywords:** Recurrent events, Asthma, Epilepsy, Calibration, Model performance

## Abstract

**Background:**

Many chronic conditions, such as epilepsy and asthma, are typified by recurrent events—repeated acute deterioration events of a similar type. Statistical models for these conditions often focus on evaluating the time to the first event. They therefore do not make use of data available on all events. Statistical models for recurrent events exist, but it is not clear how best to evaluate their performance. We compare the relative performance of statistical models for analysing recurrent events for epilepsy and asthma.

**Methods:**

We studied two clinical exemplars of common and infrequent events: asthma exacerbations using the Optimum Patient Clinical Research Database, and epileptic seizures using data from the Standard versus New Antiepileptic Drug Study. In both cases, count-based models (negative binomial and zero-inflated negative binomial) and variants on the Cox model (Andersen-Gill and Prentice, Williams and Peterson) were used to assess the risk of recurrence (of exacerbations or seizures respectively). Performance of models was evaluated via numerical (root mean square prediction error, mean absolute prediction error, and prediction bias) and graphical (calibration plots and Bland–Altman plots) approaches.

**Results:**

The performance of the prediction models for asthma and epilepsy recurrent events could be evaluated via the selected numerical and graphical measures. For both the asthma and epilepsy exemplars, the Prentice, Williams and Peterson model showed the closest agreement between predicted and observed outcomes.

**Conclusion:**

Inappropriate models can lead to incorrect conclusions which disadvantage patients. Therefore, prediction models for outcomes associated with chronic conditions should include all repeated events. Such models can be evaluated via the promoted numerical and graphical approaches alongside modified calibration measures.

**Supplementary Information:**

The online version contains supplementary material available at 10.1186/s41512-025-00187-7.

## Background

People with long-term, chronic, medical conditions often have repeated acute deteriorations of a similar type, such as seizures in epilepsy, exacerbations in asthma, or flares of inflammatory bowel disease. These recurrent events are the major drivers of morbidity and mortality for these conditions. They also lead to substantial healthcare costs and drive indirect costs such as loss of income. When these events are studied, researchers usually only include counts of events that occur prior to treatment or prior to a change in treatment for example. Consequently, they do not use all the available event information [1]. Although randomised controlled trials and observational studies for chronic conditions usually collect considerable information about individuals’ event patterns over a period of time, frequently these studies are focussed on time-to-event outcomes such as the standard internationally recognised outcomes in epilepsy of time to 12-month remission and time-to-treatment failure [2]. This means a further loss of important temporal information.

Statistical models can estimate future events for an individual, conditional on their values of multiple predictors (prognostic or risk factors) such as age, sex and biomarkers [3]. Many such models (prognostic prediction models) are published in the medical literature each year [4], and they are usually developed using a regression framework such as logistic or Cox models.

Recurrent event models, alongside dynamic and joint prediction models, are increasingly being developed to estimate the chances of a particular outcome for relevant individuals over a prolonged horizon of opportunity. However, infrequent events and common events might require different models, for example people with asthma tend to have a lower event rate than those with epilepsy and thus different models might be required.

Model performance of prediction models is traditionally assessed using discrimination and calibration [5]. Calibration refers to an agreement between observed outcomes and predictions. Discrimination refers to the ability of the prognostic model to differentiate between those who experience the event during the study and those who do not [5]. Whilst it is known that the discrimination and calibration of any prediction model should be assessed prior to use in clinical practice [5], is it not clear how to best evaluate the statistical performance of statistical models for recurrent events. This manuscript therefore compares the relative performance of statistical models for analysing recurrent events for epilepsy and asthma.

## Methods

Asthma and epilepsy are used as exemplars with less frequent and more frequent event rates respectively. R 4.4.0. statistical software has been used throughout [6].

### Datasets

#### Asthma

The Optimum Patient Care Research Database (OPCRD) comprises anonymous data from over 600 UK general practices across England, Scotland, Wales and Northern Ireland and was approved for clinical research by the Health Research Authority of the UK NHS (REC reference: 15/EM/0150). The study population consisted of all patients aged 12–80 with a Read code for an asthma diagnosis prior to the study start who were registered with a GP during the study period. Only patients prescribed regular asthma treatment on more than one occasion (i.e. not short-acting bronchodilator alone) were included in the study population for those with active asthma. Exclusion criteria were a COPD diagnostic Read code at any time, a Read code for resolved asthma during the study, or less than 3 years of data. The data comprise three consecutive yearlong observation windows. Data was collated between 2005 and 2013, with patients entering the study either at study commencement (1st January 2005) or the first point thereafter when they joined a practice participating in OPCRD, or their practice joined OPCRD.

The primary endpoint of asthma exacerbation was defined in accordance with European Respiratory Society/American Thoracic Society criteria [7], namely an asthma-related hospitalisation, emergency department (ED) attendance, or an acute respiratory presentation resulting in a course of oral corticosteroids (OCS). Events within 2 weeks were assumed lack of resolution of the initial exacerbation.

### Epilepsy

Full details of the Standard Versus New Antiepileptic Drug (SANAD) studies are available in the original trial reports [8, 9]. Arm A studied focal epilepsy whilst Arm B considered generalised epilepsy. In brief, people qualified for randomisation into Arm B of the SANAD study if they had a history of two or more clinically definite unprovoked epileptic seizures in the previous year, and if the recruiting clinician regarded valproate as the better standard treatment option than carbamazepine. Participants were randomly allocated in a 1:1:1 ratio to valproate, lamotrigine or topiramate between January 12, 1999 and August 31, 2004. The two primary outcomes in SANAD were time to treatment failure and time to the first period of 12-month remission from seizures, both originally modelled using Cox’s proportional hazards model.

During the study period, 702 people were randomised yielding 104,839 post-randomisation seizures. As common in clinical practice, 30% (212) of participants had no further seizures during the observation period. This analysis involves 702 participants although predicted counts at 2 years after randomisation are based on the subset of 509 participants who have follow-up data, or a date of loss to follow-up, recorded during this period.

#### Statistical models

Methods for the fitting count and rate-based models, as well as multiple time-to-event models such as Andersen-Gill (AG) and Prentice, Williams and Peterson (PWP) models, can be found within the literature (e.g. [10, 11]). The AG and PWP models consider the time between exacerbations and were fitted using the coxph function from within the survival package in R [12]. The negative binomial and zero-inflated negative binomial models consider exacerbation counts and were fitted using the glm.nb and glm functions within the stats package in R [6]. All four models were fitted to data from each study. Both the AG and PWP models were fitted with robust standard errors via a jackknife estimate [13]. The total time variant of the PWP model was selected for both examples as this considers the individual’s total time in the study rather than just the time between events [11].

The asthma model included gender, age, presence of previous exacerbations, smoking status and year of entry into the study. This choice was based on known prognostic factors for asthma [14, 15]. The epilepsy models included gender, first-degree relative with epilepsy, age at randomisation, and annual rate of tonic–clonic seizures prior to randomisation. Again, known prognostic factors for epilepsy were used [16]. Treatment was forced into each epilepsy model as all patients were treated at randomisation. Additionally, to draw in the extreme tail of the seizure counts, the total number of seizures per patient has been capped at 2100, the 99% quantile of all the per-person seizure counts, as recommended by Royston et al. [17].

Continuous covariates were assessed for best fit via log or linear transformations and the most frequently used transformation (log) was applied across all models to aid comparison. Fractional polynomials are recommended [18], but the methodology is not currently adapted to recurrent events.

#### Model performance methods

In the absence of discrimination and calibration methods for recurrent event models, it is necessary to consider alternative ways to evaluate model performance. Therefore, in each case, model fit was assessed via numerical and graphical measures.

#### Numerical measures

Model fit was assessed via the root mean squared prediction error (RMSPE), mean absolute prediction error (MAPE) and prediction bias. All three approaches compare predicted and observed event counts obtained from the models. In all cases, smaller absolute values correspond to a better model [19]. Formulae for these statistics can be found in Appendix 1 and were manually coded in R.

### Graphical measures

#### Calibration plots

Calibration refers to how closely the probability of the event predicted by the model agrees with the observed probability of the event within the dataset and can be assessed graphically [20]. As event probabilities are meaningless for the prediction models built using variants on the Cox model, observed event counts were shown on the x-axis and predicted event counts on the *y*-axis. Calibration plots were manually drawn in R.

### Bland–Altman plots

Bland–Altman plots are scatter plots, in which the y-axis shows the difference between the predicted and observed event count, and the *x*-axis represents the mean of the measures. They were drawn manually in R. Bland and Altman recommended setting limits of agreement at 1.96 standard deviations on either side of the mean difference, between which 95% of the difference may be expected to lie [21].

## Results

### Demographic data

Information regarding patient characteristics can be seen in Table 1 (asthma) and Table 2 (epilepsy). The specific subset of the OPCRD dataset for this analysis contained 155,163 individuals from 544 GP practices. The SANAD arm B dataset contained 702 participants. Only smoking status within OPCRD included missing data.

**Table 1:** Characteristics of 155,163 people with asthma (OPCRD)

Variable		Column %	Number of exacerbations during observation period, row %		
			0 (49%)	1 (23%)	≥ 2 (28%)
Gender	Female	57	45	23	32
	Male	43	56	21	23
Previous exacerbations	No	33	59	20	21
	Yes	67	45	23	32
Smoking status	Non-smoker	57	52	22	26
	Current smoker	15	46	22	32
	Ex-smoker	23	47	22	31
	*Missing*	*5*	*47*	*22*	*31*
Year of entry into study	2005	3	44	22	34
	2006	7	41	23	36
	2007	3	42	22	36
	2008	10	51	22	27
	2009	12	53	22	25
	2010	10	50	22	28
	2011	22	50	22	28
	2012	24	50	22	28
	2013	10	51	22	27
Age at entry into study, median (interquartile range)			44 (29, 58)	46 (33, 60)	50 (37, 63)

**Table 2:** Demographic data for arm B of SANAD

Variable		Valproate (*n* = 234)	Lamotrigine (*n* = 235)	Topiramate (*n* = 233)	Total (*n* = 702)
Gender	Female	93 (40)	95 (40)	93 (40)	281 (40)
	Male	141 (60)	140 (60)	141 (60)	422 (60)
Primary relative with epilepsy	Absent	197 (84)	183 (78)	195 (84)	575 (82)
	Present	37 (16)	52 (22)	38 (16)	127 (18)
Age at randomisation (years), median (IQR) [range]		18.9 (12.5, 27.6) [5.0, 76.9]	18.6 (12.8, 29.1) [5.3, 77.1]	18.9 (12.5, 27.6) [5.0, 75.2]	18.9 (12.5, 28.3) [5.0, 77.1]
No seizures during follow-up		82 (35)	58 (25)	72 (31)	212 (30)
Tonic–clonic seizures	Annual seizure rate prior to randomisation % < 1 seizure/year	60.8	51.5	31.8	48.0
	Median (IQR) for those with rate ≥ 1 seizure/yr	53 16.7 (4.4, 50.5)	35 21.5 (4.0, 63.7)	44 16.3 (3.5, 68.4)	44 17.95 (3.9, 63.2)

*IQR* inter-quartile range

Based on Table 1, women and smokers have more exacerbations. Additionally, previous exacerbations indicate more future exacerbations and from 2008, less than a quarter of asthma patients had an exacerbation recorded in 3 years of observation. Indeed, there were relatively few patients included for the first 3 years. According to Table 2, the characteristics of patients are similar across the three randomised treatments except for the annual seizure rate prior to randomisation. There is no clinical reason for this variation as patients were randomly assigned to the treatments.

**Fig. 1:**
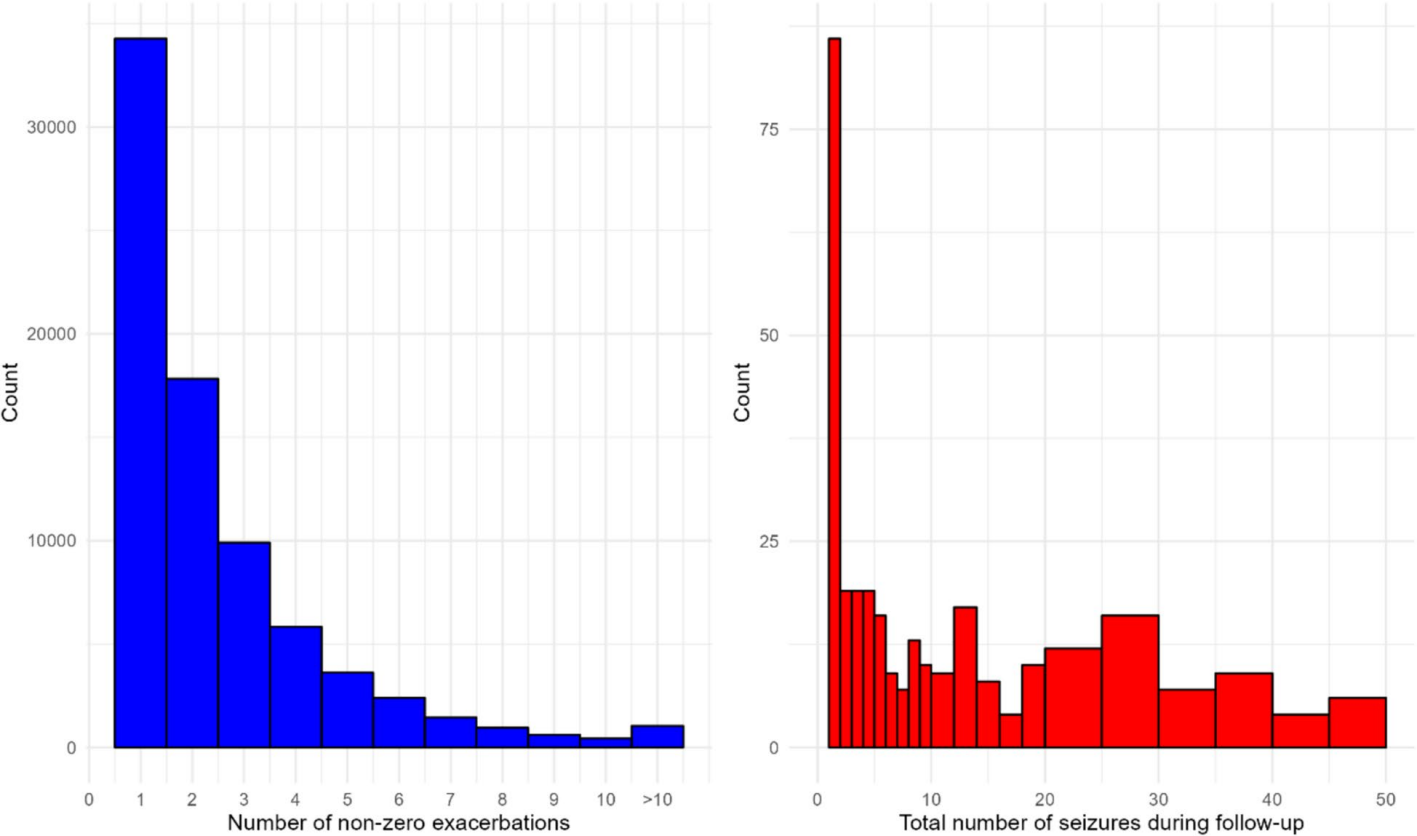
(Left) Histogram for number of exacerbations in people who had at least one asthma exacerbation. (Right) Histogram showing the number of non-zero seizures reported per person during follow-up (capped at 50)

Over the 3-year observation period, 49% (76,801) of individuals did not experience an asthma exacerbation. Of those that did, 34,277 had one (44%). For people with asthma who were having exacerbations, the median was 3 exacerbations with an IQR of 2 to 4 (Fig. 1, left).

Based on the epilepsy data, over the duration of the study, 30% (212) of individuals did not experience seizures. A histogram of the number of non-zero seizures reported during follow-up, per patient with epilepsy, can be seen in Fig. 1 (right)—it is capped at 50 although people did report between 0 and 2100 seizures during the observation period. The median number of non-zero seizures per person was 20 (interquartile range 4–150).

### Covariate effects

Data for 147,908 individuals with asthma but without missing data for smoking status were used to fit all four multivariable models—negative binomial (exacerbation count), zero-inflated negative binomial (exacerbation count), Andersen-Gill (time between exacerbations) and PWP (time between exacerbations). The results can be seen in Table 3.

**Table 3:** Effects of covariates on exacerbation occurrence or rate (OPCRD)

Variable	Level	NB: OR (95% CI)	ZINB logit: OR (95% CI)	ZINB count: RR (95% CI)	AG: HR (95% CI)	PWP-TT: HR (95% CI)
Intercept		− 8.50 (− 8.59, − 8.41)	− 5.21 (− 5.57, − 4.86)	− 7.55 (− 7.68, − 7.42)	N/A	N/A
Age (log*)		1.41 (1.38, 1.44)	0.75 (0.69, 0.82)	1.28 (1.24, 1.32)	1.42 (1.40, 1.44)	1.30 (1.29, 1.32)
Gender	Female	1.00	1.00	1.00	1.00	1.00
	Male	0.74 (0.72, 0.75)	1.52 (1.40, 1.65)	0.82 (0.80, 0.84)	0.77 (0.76, 0.78)	0.83 (0.82, 0.84)
Previous exacerbations	No	1.00	1.00	1.00	1.00	1.00
	Yes	2.01 (1.97, 2.06)	0.32 (0.29, 0.35)	1.40 (1.35, 1.45)	1.46 (1.44, 1.49)	1.30 (1.28, 1.31)
Smoking status	Non-smoker	1.00	1.00	1.00	1.00	1.00
	Current smoker	1.32 (1.29, 1.36)	0.72 (0.65, 0.80)	1.23 (1.19, 1.27)	1.26 (1.23, 1.28)	1.18 (1.16, 1.19)
	Ex-smoker	1.13 (1.10, 1.16)	0.90 (0.82, 0.99)	1.10 (1.07, 1.14)	1.09 (1.07, 1.11)	1.06 (1.05, 1.07)
Year of entry into study	2013	1.00	1.00	1.00	1.00	1.00
	2005	1.16 (1.06, 1.28)	1.14 (0.81, 1.62)	1.21 (1.06, 1.37)	1.35 (1.27, 1.44)	1.25 (1.20, 1.31)
	2006	1.22 (1.16, 1.28)	0.71 (0.57, 0.89)	1.12 (1.05, 1.19)	1.30 (1.25, 1.34)	1.21 (1.18, 1.24)
	2007	1.27 (1.19, 1.35)	0.80 (0.62, 1.04)	1.21 (1.11, 1.30)	1.27 (1.22, 1.33)	1.18 (1.14, 1.21)
	2008	1.09 (1.05, 1.14)	0.78 (0.65, 0.93)	1.03 (0.97, 1.09)	1.05 (1.01, 1.08)	1.02 (1.00, 1.05)
	2009	1.05 (1.01, 1.10)	0.78 (0.66, 0.93)	0.99 (0.94, 1.05)	0.99 (0.96, 1.02)	0.99 (0.97, 1.01)
	2010	1.09 (1.05, 1.14)	0.83 (0.70, 0.98)	1.04 (0.99, 1.10)	1.04 (1.01, 1.07)	1.02 (1.00, 1.04)
	2011	1.05 (1.05, 1.09)	0.85 (0.73, 1.00)	1.01 (0.96, 1.06)	1.04 (1.01, 1.07)	1.02 (1.00, 1.04)
	2012	1.06 (1.03, 1.10)	1.01 (0.87, 1.16)	1.07 (1.02, 1.12)	1.01 (0.98, 1.04)	0.99 (0.97, 1.01)

*NB* negative binomial, *ZINB* zero-inflated negative binomial, *AG* Andersen-Gill, *PWP-TT* Prentice, Williams and Peterson total time, *RR* relative risk, *OR* odds ratio, *HR* hazard ratio

^*^Log transformation identified through multivariable fractional polynomial procedure where applicable

Each model requires a different interpretation. The results from the negative binomial model are the adjusted odds ratio for exacerbation count—an adjusted odds ratio of 1.32 for a current smoker for example suggests that the chance of an exacerbation is 32% more likely in people who smoke than in non-smokers when accounting for the contributions of the other predictors. Adjusted odds ratios for exacerbation count are equivalent to the adjusted odds ratios from the count component of the zero-inflated negative binomial model.

The zero-inflated negative binomial model leads to two different outputs—a logit component and a count component. The results for the count component are the adjusted relative risk of having an exacerbation—an adjusted relative risk of 1.40 for previous exacerbation for example suggests that the chance of an exacerbation is 40% higher in people with a previous exacerbation than those without when accounting for the contributions of the other predictors. The logit component is more challenging to interpret. The result is an adjusted odds ratio but rather than being the odds of having an exacerbation they are the adjusted odds of being an ‘excessive zero’. An excessive zero is defined as more zeros than expected by the distribution we are using for the modelling [22] For example, for gender, the odds ratio for the logit component of the zero-inflated negative binomial model is 1.52. Therefore, the adjusted odds of there being an excessive zero increased by 52% for men compared to women so, men have a lower chance of exacerbations than women do (when accounting for the contributions of the other predictors).

The results for the Andersen-Gill and PWP models are adjusted hazard ratios for the rate of future exacerbations—an adjusted hazard ratio of 1.46 for previous exacerbation from the Andersen-Gill model for example suggests that the chance of an exacerbation is 46% more likely in people with a previous exacerbation than in those without when accounting for the contributions of the other predictors.

Table 4 shows the contribution of each predictor within all four models fitted to the example epilepsy data. The results are fairly consistent across the four models except for the effect of gender within the logistic component of the zero-inflated model. According to these results, men are less likely to have exacerbations than women. Results from the other models suggest that women have a higher rate of seizures. Therefore, the data show that men are less likely to report a single exacerbation than women.

**Table 4:** Effects of covariates on seizure occurrence or rate (SANAD)

Variable		NB: OR (95% CI)	ZINB logit: OR (95% CI)	ZINB count: RR (95% CI)	AG: HR (95% CI)	PWP-TT: HR (95% CI)
Intercept		6.54 (5.66, 7.47)	2.23 (1.24, 3.25)	6.31 (5.583, 7.08)	N/A	N/A
Gender	Female	1.00	1.00	1.00	1.00	1.00
	Male	1.15 (0.79, 1.67)	0.60 (0.41, 0.88)	1.37 (1.00, 1.88)	1.31 (0.90, 1.91)	1.09 (1.00, 1.20)
Primary relative with epilepsy	Absent	1.00	1.00	1.00	1.00	1.00
	Present	2.10 (1.36, 3.37)	2.00 (1.21, 3.42)	1.83 (1.28, 2.68)	1.74 (1.07, 2.81)	1.09 (0.96, 1.24)
Treatment	Valproate	1.00	1.00	1.00	1.00	1.00
	Lamotrigine	1.74 (1.13, 2.69)	1.83 (1.18, 2.85)	1.52 (1.05, 2.21)	2.01 (1.27, 3.18)	1.14 (1.02, 1.27)
	Topiramate	1.55 (1.01, 2.39)	1.33 (0.87, 2.03)	1.55 (1.06, 2.26)	1.92 (1.18, 3.12)	1.17 (1.03, 1.31)
Age at randomisation (years)		0.28 (0.21, 0.37)	0.44 (0.32, 0.60)	0.34 (0.27, 0.43)	0.36 (0.24, 0.53)	0.90 (0.83, 0.98)
Annual tonic–clonic seizure rate prior to randomisation		0.86 (0.81, 0.91)	0.96 (0.89, 1.03)	0.86 (0.82, 0.90)	0.88 (0.81, 0.95)	1.02 (1.00, 1.03)

*NB* negative binomial, *ZINB* zero-inflated negative binomial, *AG* Andersen-Gill, *PWP-TT* Prentice, Williams and Peterson total time, *RR* relative risk, *OR* odds ratio, *HR* hazard ratio

### Numerical measures of model performance

Model fit statistics for the asthma and epilepsy models are presented in Table 5. Results show that for the low-event rate asthma data, the negative binomial and zero-inflated negative binomial models for exacerbation counts have high RMPSE and MAPE values although their prediction errors are small. High values for RMSPE and MAPE suggest poor model fit. The AG and PWP models have the smallest MAPE and RMPSE, showing good model fit but have large prediction errors demonstrating large uncertainty over the model fit. The PWP model has a slightly lower RMPSE than the AG model but equivalent MAPE values showing fairly similar model performance across the models.

**Table 5:** Model fit statistics based on the predictions from the five models using both example datasets

	Exacerbations (OPCRD)			Seizures (SANAD)		
Model	RMPSE	MAPE	Prediction bias	RMPSE	MAPE	Prediction bias
Negative binomial	1.25	1.35	0.11	20.70	1.23	18.43
ZINB	1.23	1.33	0.11	21.48	1.45	30.51
Andersen-Gill	0.99	0.86	0.87	26.25	1.61	− 48.01
PWP	0.95	0.86	0.87	1.79	0.51	− 3.15

For the high-event rate epilepsy data, according to RMPSE, MAPE and prediction bias, the PWP model is the best fit for the data as all three metrics had small values. The differences between the observed and predicted seizure counts are much smaller than for the other three models. The inclusion of the zero-inflation term gives a model which substantially overestimates counts, more than the standard negative binomial distribution.

### Graphical measures

#### Calibration plots

**Fig. 2:**
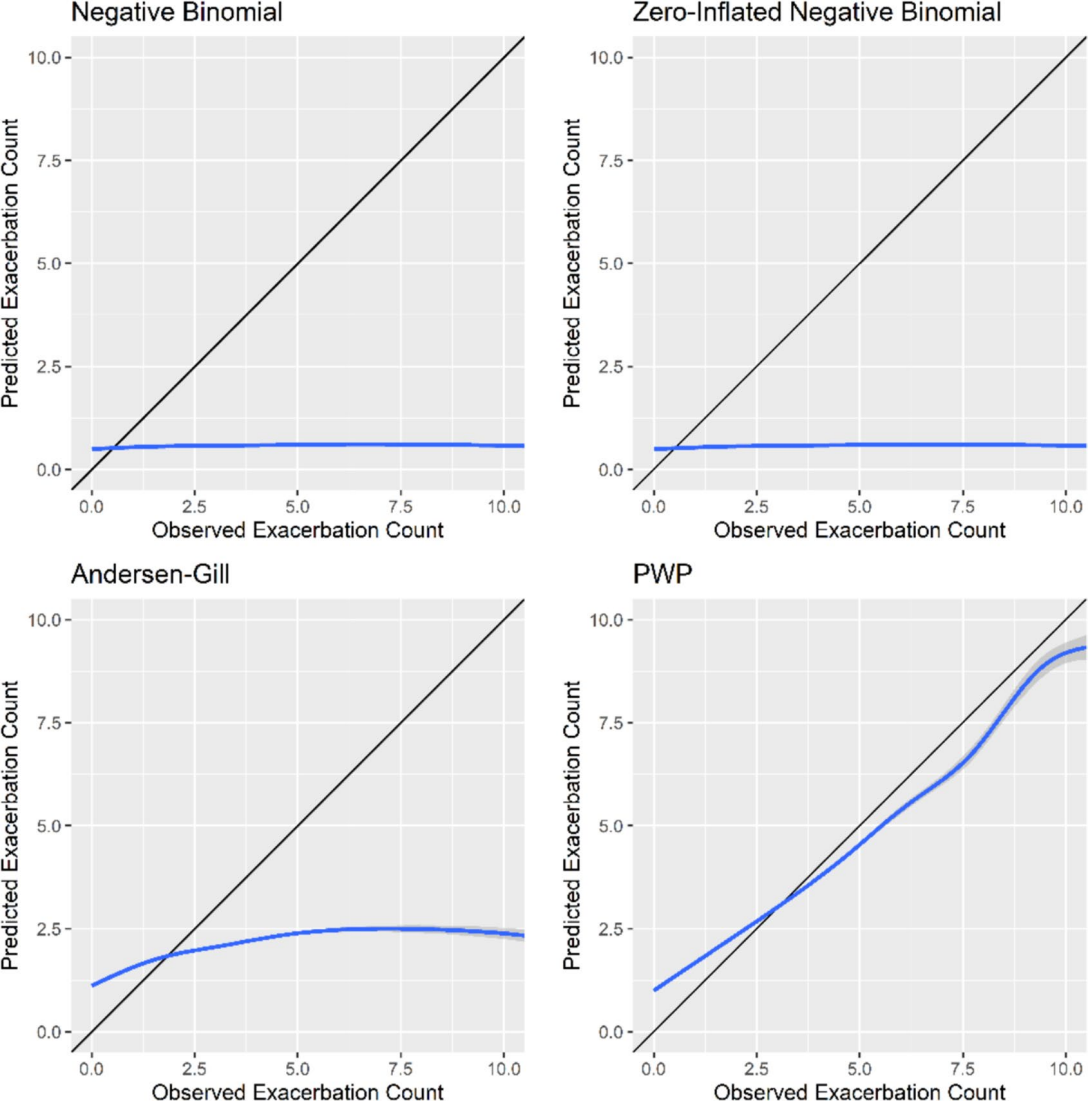
Calibration plots comparing observed and predicted asthma exacerbation counts

Calibration plots of observed and predicted exacerbation counts for the four asthma models show that the PWP model fits quite well, and the other three fail (Fig. 2). The PWP plot has a smooth-fitted line fairly close to the 45° line of agreement. The negative binomial, zero-inflated negative binomial and Andersen-Gill models have fitted lines far from agreement.

**Fig. 3:**
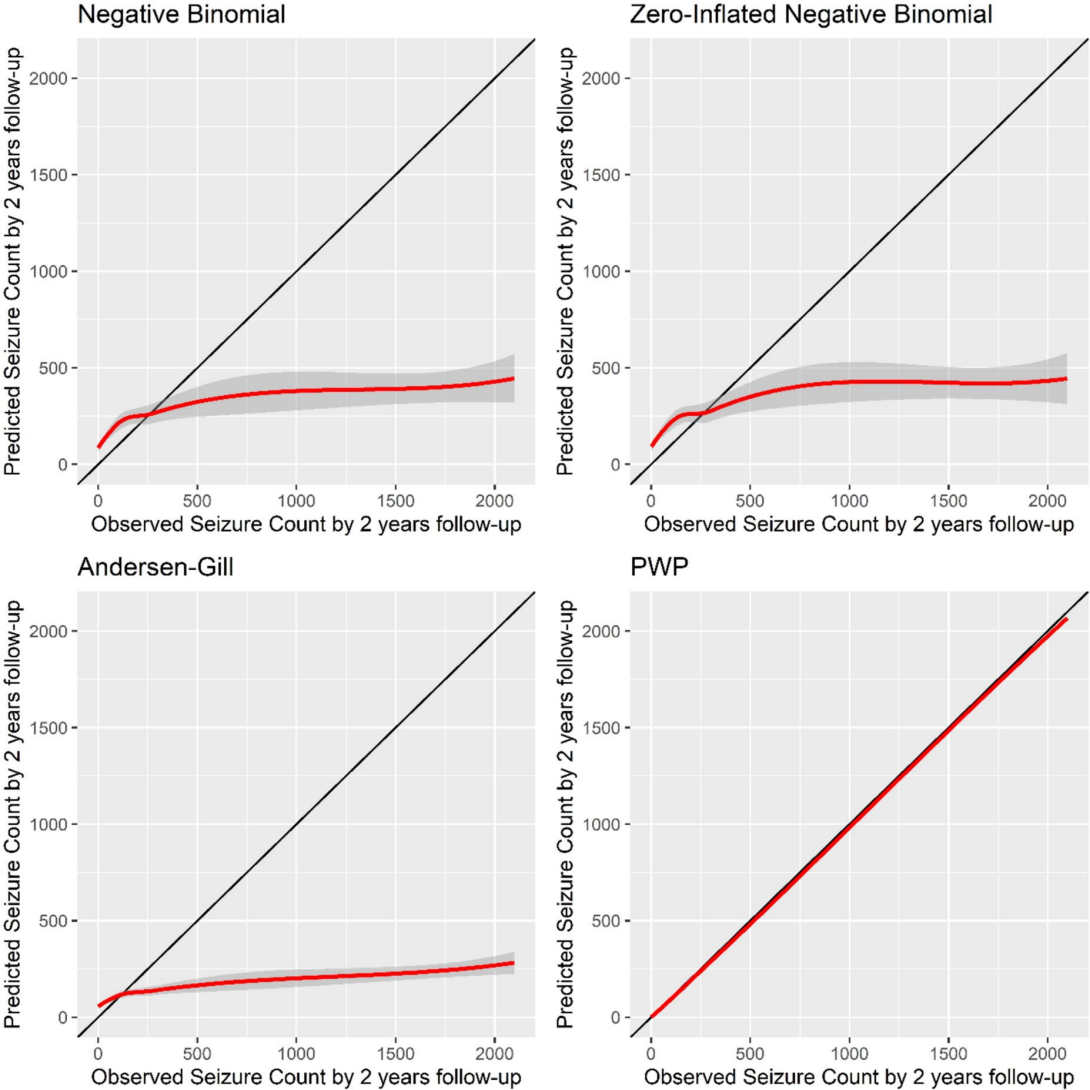
Calibration plots comparing observed and predicted seizure counts

According to the calibration plots for the epilepsy example (Fig. 3), the PWP model shows excellent agreement between the observed and predicted seizure counts. The negative binomial, zero-inflated negative binomial and Andersen-Gill models are clearly inadequate for prediction. The calibration plots suggest that these models predict up to 250 seizures with tighter confidence bands than predictions beyond 250 seizures. This may be because of the diversity of the observed seizure counts. Conversely, the PWP-TT model demonstrates tight confidence bands irrespective of the number of observed or predicted seizures, which suggests that it is using the data more efficiently than the other models.

### Bland–Altman plots

#### Asthma

Given the low event rate for the asthma plot, it is not appropriate to draw Bland–Altman plots as this method provides only limited information when the range of observed values is small relative to the number of observations [23]. Instead, deviance residuals were considered. Deviance residuals are numerical measurements of agreement between a model’s fit and the fit of an ideal model. If a deviance residual is 0, it indicates that the value for that data point is identical to the value for that data point in the ideal model and thus the model fits the data well [24]. The associated formula is presented in Appendix 2.

Tables of deviance residuals were examined to determine if any non-fitting was systematic across patient characteristics. Prediction bias according to combinations of risk factors can be seen in Table 6. The values summarise how far, on average, the predicted counts are from the actual counts. The zero-inflated negative binomial is the least biased, with a slightly smaller prediction bias than the negative binomial. The predictions for people with previous exacerbations are less accurate than for those without.

**Table 6:** Z-score statistics based on the predictions from the four models

Prediction bias (*n*)	Non-smoker	Current smoker	Ex-smoker
Negative binomial			
Female, previous exacerbations	0.17	0.21	0.21
Male, previous exacerbations	0.12	0.24	0.14
Female, no previous exacerbations	− 0.02	− 0.03	0.02
Male, no previous exacerbations	0.01	0.05	0.00
Zero-inflated negative binomial			
Female, previous exacerbations	0.16	0.18	0.19
Male, previous exacerbations	0.12	0.24	0.14
Female, no previous exacerbations	− 0.01	− 0.01	0.03
Male, no previous exacerbations	− 0.01	0.04	−0.01
Andersen-Gill			
Female, previous exacerbations	1.07	1.34	1.25
Male, previous exacerbations	0.72	0.96	0.91
Female, no previous exacerbations	0.61	0.77	0.75
Male, no previous exacerbations	0.43	0.57	0.54
PWP			
Female, previous exacerbations	1.06	1.39	1.25
Male, previous exacerbations	0.73	0.92	0.91
Female, no previous exacerbations	0.61	0.76	0.73
Male, no previous exacerbations	0.44	0.56	0.55

#### Epilepsy

**Fig. 4:**
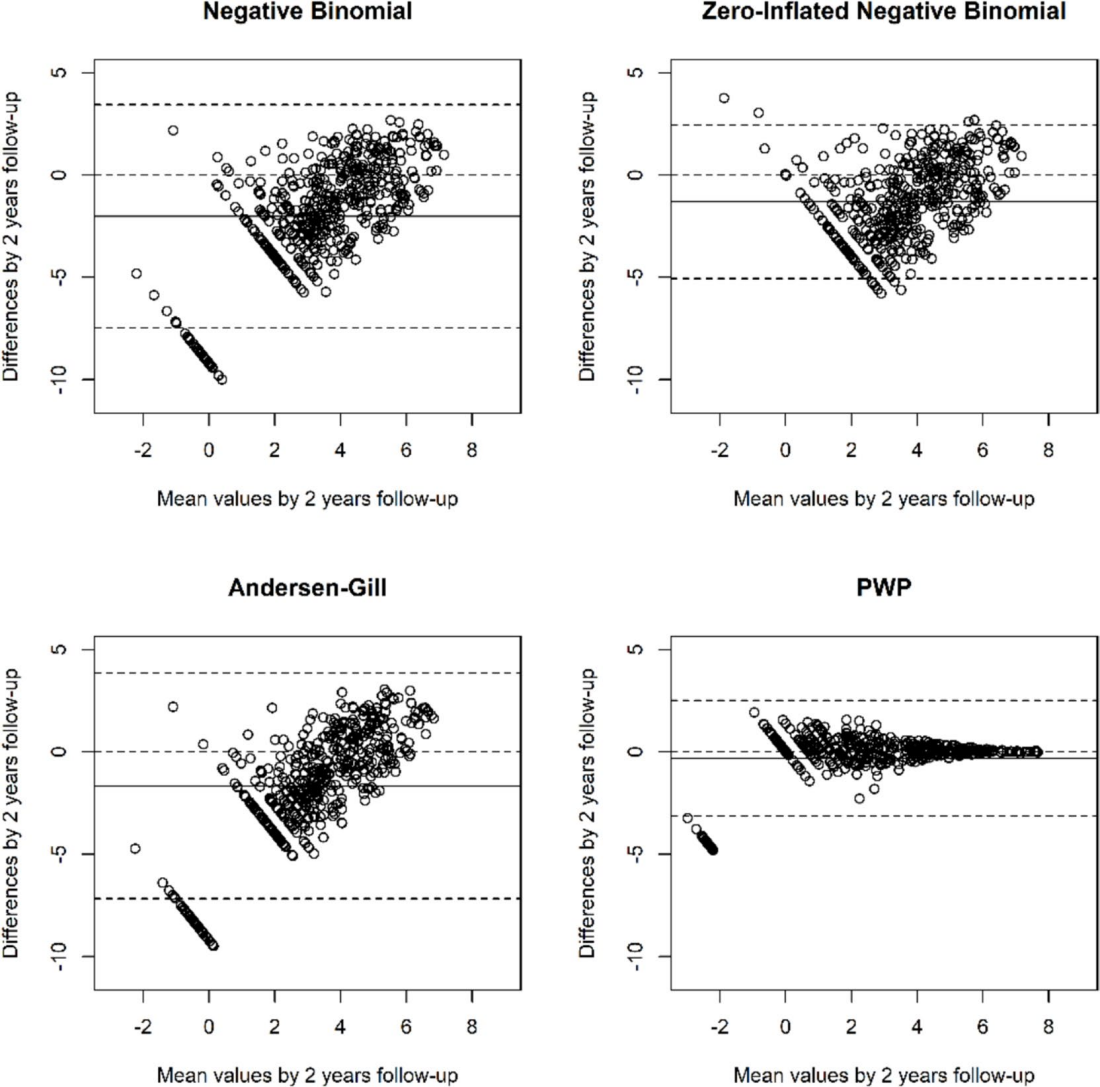
Bland–Altman style plots by 2 years after randomisation comparing the mean of the predicted and observed seizure counts by the difference between the observed and predicted counts

The Bland–Altman plots for the epilepsy dataset can be seen in Fig. 4. The solid black line is the mean of the differences and the dotted lines represent a 95% limit of agreement around the mean difference. The discrepancy between the observed and expected counts is smaller for the PWP-TT model than the other three suggesting that this model fits the data better than the other three. Additionally, the limits of agreement are much narrower for the PWP-TT model than the other three suggesting that the predictions are in close agreement from this model. The small group of patients at the left (mean about − 2) have higher observed counts than predicted by the PWP-TT models.

## Discussion

This analysis considered numerical and graphical ways to evaluate the model performance of recurrent event prediction models. Examples of asthma and epilepsy datasets were modelled in four ways—negative binomial, zero-inflated negative binomial, Andersen-Gill and PWP. Numerical and graphical measures determined that the PWP model was the most appropriate model for the asthma and epilepsy examples as the predicted event counts from this model most closely matched the observed counts. However, the negative binomial did show much better performance for the asthma dataset than the epilepsy dataset and thus may be more suited to low event rate data.

Few published analyses of clinical data have utilised statistical models for recurrent event data. Those that have included an analysis of diarrhoeal episodes in children [25], a population-based study of repetitive traumatic brain injury among persons with traumatic brain injury [26], recurrent malaria episodes [27], and childhood infectious diseases [28]. Whilst these publications demonstrate a comparison of approaches, there is relatively little information or practical guidance on how to evaluate the performance of the fitted models.

This paper is the first to successfully apply existing methods to evaluate the performance of prediction models for recurrent event data. By choosing two different clinical areas typified by different underlying event rates, and four different modelling approaches, we have been able to highlight the benefits and shortcomings of a variety of approaches. Whilst some model choices will be informed by the available data (event counts or dates of events for example) and assumptions regarding the independence of the events [11], this is the first analysis to consider approaches to evaluate the performance of statistical models for recurrent events.

There are limitations to this analysis. First, the outcome measures agreed upon by the International League Against Epilepsy [22] combined with how dates of seizures are collected in randomised controlled trials and clinical practice for people with epilepsy necessitates the imputation of event times. Some event-specific times are unavailable within the epilepsy dataset. Imputation, assuming a constant event rate, has been used. Where there were no events, no event time has been imputed and if there were only one or two seizures between visits the dates of these seizures will be recorded exactly.

SANAD was a randomised controlled trial that may be considered to only include a highly selected patient cohort with a tightly controlled ecology of care. This may potentially have led to an overestimation of predicted event counts across the models. The opposite is true for OPCRD. It is real-world data so there will be some individuals included who do not actually have asthma and some events that are not captured. This will increase the noise within the dataset but is unlikely to bias the results in any given direction.

The varied number of events per individual, particularly for epilepsy means that some strata of the PWP model have only a few events which could lead to an over-optimistic impression of the PWP. Despite this, the results suggest that the PWP model fits the epilepsy data better than the other models. For asthma, with none or few events over 3 years, the zero-inflated negative binomial model may be most appropriate as there are likely too few events to reliably estimate the rates used in the AG and PWP models [11].

Work is now needed to develop a methodology to evaluate the discrimination of recurrent event prediction models, as required by the Transparent reporting of a multivariable prediction model for individual prognosis or diagnosis (TRIPOD) reporting guidelines [29, 30]. Additionally, only two clinical examples have been considered here. Whilst they have different underlying event rates it is important to evaluate which recurrent event models should be used given the underlying clinical event rate via a simulation study for example. Finally, it will be important to develop software packages to support the development and evaluation of model performance of recurrent event prediction models.

## Conclusions

Usually, data involving recurrent conditions are modelled by selecting an endpoint at a fixed time point of interest such as time to first asthma exacerbation after diagnosis or time 12-month remission from seizures after commencing treatment for people with epilepsy. Although this is relatively simple to analyse, valuable information about the participant’s event journey is lost. There are several alternatives including the negative binomial, zero-inflated negative binomial, Andersen-Gill and PWP models. However, there is limited guidance as to how to evaluate the performance of such models. This study highlights the potential of straightforward evaluation techniques to highlight marked differences in the performance of available models when analysing clinical datasets.

This work has the potential to improve the way that chronic conditions typified by recurrent events are modelled in the future. In turn, this may lead to more appropriate clinical prediction models and therefore improved treatment choice and patient counselling.

## Supplementary Information

Additional file 1: Appendix 1. The RMPSE, MAPE and bias are calculated as shown in Eqs. 1–3 respectively. In each equation n is the number of patients in the dataset, yi is the observed event count for patient i, and y^i is the predicted event count for patient i. Appendix 2. The formula for calculating deviance residuals varies across statistical models. However, it can be generalised as shown in Eq. 4 where D is the deviance, di are the deviance residuals and represents the log likelihood. It is a readily available output in all statistical software packages following fitting of a statistical model.

## Data Availability

OPCRD data may be obtained from a third party and are not publicly available. The SANAD datasets analysed during the current study are not publicly available as they contain information that could comprise the privacy of participants but are available from the Professor Marson (A.G.Marson@liverpool.ac.uk) on reasonable request.
