# Supplementary material for: Guide to evaluating performance of prediction models for recurrent clinical events
Source: Diagn Progn Res. 2025 Mar 17;9:6. doi: 10.1186/s41512-025-00187-7 (PMC11912649; doi:10.1186/s41512-025-00187-7)
Supplement: Supplementary file 1 — Additional file 1: Appendix 1. The RMPSE, MAPE and bias are calculated as shown in Eqs. 1–3 respectively. In each equation n is the number of patients in the dataset, yi is the observed event count for patient i, and y^i is the predicted event count for patient i. Appendix 2. The formula for calculating deviance residuals varies across statistical models. However, it can be generalised as shown in Eq. 4 where D is the deviance, di are the deviance residuals and represents the log likelihood. It is a readily available output in all statistical software packages following fitting of a statistical model. [file 41512_2025_187_MOESM1_ESM.pdf]

Additional File 1

Appendix 1

The RMPSE, MAPE and bias are calculated as shown in Equations 1-3 respectively. In each equation n is the number of patients in the dataset, y_i is the observed event count for patient i, and $\hat{y}_i$ is the predicted event count for patient i.

Equation 1

$$RMSPE = \sqrt{\frac{1}{n}\sum_i \frac{(\hat{y}_i - y_i)^2}{\hat{y}_i}}$$

Equation 2

$$MAPE = \frac{1}{n}\sum_i \frac{|\hat{y}_i - y_i|}{\hat{y}_i}$$

Equation 3

$$Bias = \frac{1}{n}\sum_i \hat{y}_i - \frac{1}{n}\sum_i y_i$$

To calculate the expected number of seizures according to the Andersen-Gill and PWP models, the baseline hazard has been estimated. This has then enabled calculation of absolute predictions within any specified time frame, rather than relative predictions.(1)

Appendix 2

The formula for calculating deviance residuals varies across statistical models. However, it can be generalised as shown in Equation 4 where D is the deviance, d_i are the deviance residuals and L represents the log likelihood.(2) It is a readily available output in all statistical software packages following fitting of a statistical model.

Equation 4

$$D = 2\big(L(idealmodel) - L(fittedmodel)\big) = \sum_{i}^{n} d_i^2$$

To identify whether there are systematic differences in the model fit across the characteristics the median deviance residual according to clinical characteristics of interest should be estimated. The comparison of deviance residuals across models is appropriate given the approximation of the log-likelihood deviance residual to a χ^2 distribution.(3)

1. Therneau TM, Grambsch PM. Modeling Survival Data: Extending the Cox Model: Springer New York; 2013.
2. Altman DG. Practical statistics for medical research. London: Chapman & Hall; 1991.
3. McCullagh P. Generalized linear models: Routledge; 2018.
